# Hybrid inorganic-organic capsules for efficient intracellular delivery of novel siRNAs against influenza A (H1N1) virus infection

**DOI:** 10.1038/s41598-017-00200-0

**Published:** 2017-03-07

**Authors:** Alexander S. Timin, Albert R. Muslimov, Aleksandra V. Petrova, Kirill V. Lepik, Maria V. Okilova, Andrey V. Vasin, Boris V. Afanasyev, Gleb B. Sukhorukov

**Affiliations:** 1 RASA center in Tomsk, Tomsk Polytechnic University, Lenin Avenue, 30, 634050 Tomsk, Russian Federation; 2 First I. P. Pavlov State Medical University of St. Petersburg, Lev Tolstoy str., 6/8, 197022 Saint-Petersburg, Russian Federation; 3 RASA center in St. Petersburg, Peter The Great St. Petersburg Polytechnic University, Polytechnicheskaya, 29, 195251 St. Petersburg, Russian Federation; 4 Research Institute of Influenza, Popova str., 15/17, 197376 Saint-Petersburg, Russian Federation; 5 Department of Molecular Biology, Peter The Great St. Petersburg Polytechnic University, Polytechnicheskaya, 29, 195251 St. Petersburg, Russian Federation; 6 School of Engineering and Materials Science, Queen Mary University of London, Mile End Road, E1 4NS London, UK

## Abstract

The implementation of RNAi technology into the clinical practice has been significantly postponing due to the issues regarding to the delivery of naked siRNA predominantly to target cells. Here we report the approach to enhance the efficiency of siRNA delivery by encapsulating the siRNA into new carrier systems which are obtained via the combination of widely used layer-by-layer technique and *in situ* modification by sol-gel chemistry. We used three types of siRNAs (NP-717, NP-1155 and NP-1496) in encapsulated form as new therapeutic agents against H1N1 influenza virus infection. By employing the hybrid microcontainers for the siRNA encapsulation we demonstrate the reduction of viral nucleoprotein (NP) level and inhibition of influenza virus production in infected cell lines (MDCK and A549). The obtained hybrid carriers based on assembled biodegradable polyelectrolytes and sol-gel coating possess several advantages such as a high cell uptake efficiency, low toxicity, efficient intracellular delivery of siRNAs and the protection of siRNAs from premature degradation before reaching the target cells. These findings underpin a great potential of versatile microencapsulation technology for the development of anti-viral RNAi delivery systems against influenza virus infection.

## Introduction

Influenza viruses are a significant cause of morbidity and mortality worldwide and can be considered as one of the actual problem of international healthcare system. For humans, the influenza type A is the main cause for infection outbreaks and pandemics throughout history^1^. At least 50 million people in various parts of the world have died from “Spanish Flu” pandemic that has took place between 1918–1919. Major influenza A pandemics occurred in 1957 (“Asian influenza”) and 1968 (“Hong Kong influenza”) caused significant global morbidity and mortality. According to the World Health Organization (WHO), it was registered around 608 laboratory-confirmed human cases of H5N1 avian influenza for the period from 2003 to 2012^2^. A new human influenza pandemic could cause 20% of the global population to become affected by this infection^3^. Therefore, the development of new approaches for the efficient treatment of influenza viruses is very important.

At the moment, four licensed influenza antiviral prescription drugs can be used for treatment or prevention of influenza^4^. Oseltamivir and zanamivir are chemical antiviral medications against influenza A and B viruses while amantadine and rimantadine are antiviral drugs against only influenza A viruses. However, as influenza virus undergoes mutation very rapidly and, therefore, can evolve drug resistance, the efficiency of antiviral drugs can be decreased during a flu outbreak^5, 6^. Due to the possibility of new influenza pandemic, current vaccines and antiviral drugs might be ineffective and new approaches for the treatment influenza viruses should be considered. One of the most perspective approaches of antiviral treatment is based on the application of RNAi technology. RNAi is a naturally occurring process of inhibition of gene expression which is closely connected with antiviral immunity. RNAi response is activated when short dsRNAs (~21 nucleotides in length), called short interfering RNAs (siRNAs) is incorporated into the enzymatic RNA induced silencing complex (RISC), where a helicase unwinds the duplex siRNA. The resulting antisense strand guides the RISC associated nuclease to its complementary mRNA, which will be cleaved^7^, preventing the synthesis of protein. The advantages of such RNAi therapeutics is their ability to target genes with great sequence homology.

The influenza A viruses are negative-sense, single stranded RNA viruses of orthomyxoviridae family. Influenza A genome consists of eight segments, which encodes 11 proteins^8^. The viral polymerase, consisting of three subunits, PA, PB1, and PB2, is responsible for replication and transcription, and hemagglutinin (HA) and neuraminidase (NA) are critical for viral entry and release. Following initial interaction of its HA with its N-acetylneuraminic (sialic) acid receptor on the cell surface, the virus enters the cell by receptor-mediated endocytosis. Upon endosomal acidification, the HA protein undergoes conformational changes and mediates fusion between the viral envelope and endosomal membrane. The acidic environment of the endosome also triggers the disassembly of the viral core and the release of the viral ribonucleoprotein (rNP) into the cytoplasm of the cell^9^. Then, the rNPs are rapidly imported into the nucleus, catalyzing the viral genome replication and RNA transcription^10^. Subsequently, newly formed rNPs, in association with other viral proteins are exported into the cytoplasm and transported to the cell membrane for budding and release. Almost every step and components of this complex process of virus replication may be targeted with RNAi. The efficiency of such approach was demonstrated in several *in vitro* and *in vivo* models. Moreover, the application of siRNAs has been effectively demonstrated in the treatment of animals against pathogenic avian influenza A viruses of the H5 and H7 subtypes^11–14^.

Despite the great potential of RNAi technology, the main drawbacks that prevent the implementation of siRNAs into the clinical practice is associated with the problem of delivery. Due to the negative charge of RNA molecule, it is unable to be internalized into the cell cytoplasm. At the same time, it is rapidly degraded in biological fluids by endo- and exonucleases^15^. Several methods have been developed for *in vitro* and *in vivo* delivery of genetic materials, including siRNA^16–18^. Among them, the most effective is to use special carriers. But, for *in vitro* and *in vivo* application of carriers to deliver siRNA, three major criteria should be fulfilled. Such carrier should protect siRNA in intercellular matrices (biological fluids) against nucleases, provide transport across biological barriers (the plasma membrane) and exclude toxic or off-target effects. Frequently used non-viral carriers include lipoplexes and polyplexes^19^. Moreover, there are several commercially available carriers based on different cationic polymers such as polyethylenimine (PEI), chitosan (CS), poly(L-lysine) (PLL), and poly(allylamine) (PAA)^20, 21^. Apart from lipoplexes and polyplexes, polymeric or inorganic nano- and microparticles are currently being discussed as non-viral carriers for the delivery of siRNAs^22–24^.

Among the diversity of carriers, polyelectrolyte (PE) microcapsules prepared by the layer-by-layer (LbL) technique had been demonstrated as an unique tool for *in situ* encapsulation of the genetic materials to perform highly efficient *in vitro* delivery^25–30^. These capsules have many attributes that lend to their application in biomedicine. They are assembled under native conditions so that biologically active molecules are not chemically altered or inactivated. The meshwork of polymers formed in layer construction results in a structure that is permeable to small molecules. So an obvious use of microcapsules is as a delivery vehicle, and their permeable structure means they can act as a depot for release and they allow to control release of therapeutic agents at targeted places and at a desired time upon internal or external stimuli^31–36^. Also, incorporating metal nanoparticles in the microcapsule structure provides a target for heating using a laser, ultrasound or a high frequency alternating magnetic field, resulting in cargo release^37–39^. Nowadays, the use of sol-gel chemistry for the functionalization of PE microcapsules is a particular attractive aspect that could facilitate the development of PE microcapsules in biomedicine. Indeed, the sol-gel chemistry provides excellent opportunity to fabricate hybrid microcapsules with novel physicochemical features, including a low permeability and intracellular degradation^40, 41^. Here, we focus on biodegradable polyelectrolytes and orthosilicate as sol-gel precursor for the formation of SiO_2_ shell onto the surface of microcapsule. Introduction of SiO_2_ nanostructures could significantly improve mechanical strength and stability of formed hybrid materials. Besides, the use of SiO_2_ can enhance the bioactive behavior of modified materials, due to the possibility to be easily internalized into cells and SiO_2_ can be dissolved in biological environments^42^.

In this work, we explored the combination of sol-gel coating with LbL method to enhance encapsulation efficiency of siRNAs. Such hybrid capsules possess new morphological features and improved mechanical properties that protect from the decomposition of siRNA before reaching the target cells. To examine capsule internalization and cellular localization of siRNA, siRNA labeled by fluorescent marker was used. The uptake efficiency and cellular localization of fluorescently labeled siRNA was monitored using confocal laser scanning microscopy and flow cytometry. We demonstrated the feasibility for *in vitro* delivery of anti-viral siRNAs (NP-1155, NP-717 and NP-1496) using novel hybrid microcontainers. The reduction of viral nucleoprotein (NP) level and the inhibition of virus titers were observed after H1N1 virus infection in MDCK and A549 cells treated by different concentration of siRNA delivered via hybrid microcontainers. This study shows that microencapsulation technology for the delivery of antiviral siRNA is an efficient tool for silencing influenza virus infection and in development of siRNA delivery approach for combating viral infection diseases in humans.

## Results and Discussion

### Preparation of SiO_2_-coated hybrid capsules and their characteristics

The developing new strategy for siRNA delivery showing in virus gene silencing and a reduction of viral production is highly desirable. So, we examine the potency of novel microcontainers for delivery of antiviral siRNAs. We divided our research into several steps, including fabrication of hybrid microcarriers, evaluation of loading capacity for siRNA, capsules internalization, their toxicity and effect of encapsulated antiviral siRNAs on the inhibition of viral nucleoprotein and virus titer.

**Figure 1:**
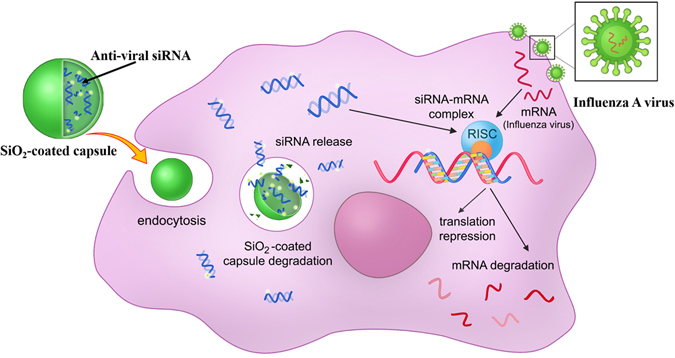
The schematic illustration of the intracellular delivery of antiviral siRNA against influenza A virus using SiO_2_-coated hybrid capsules.

**Figure 2:**
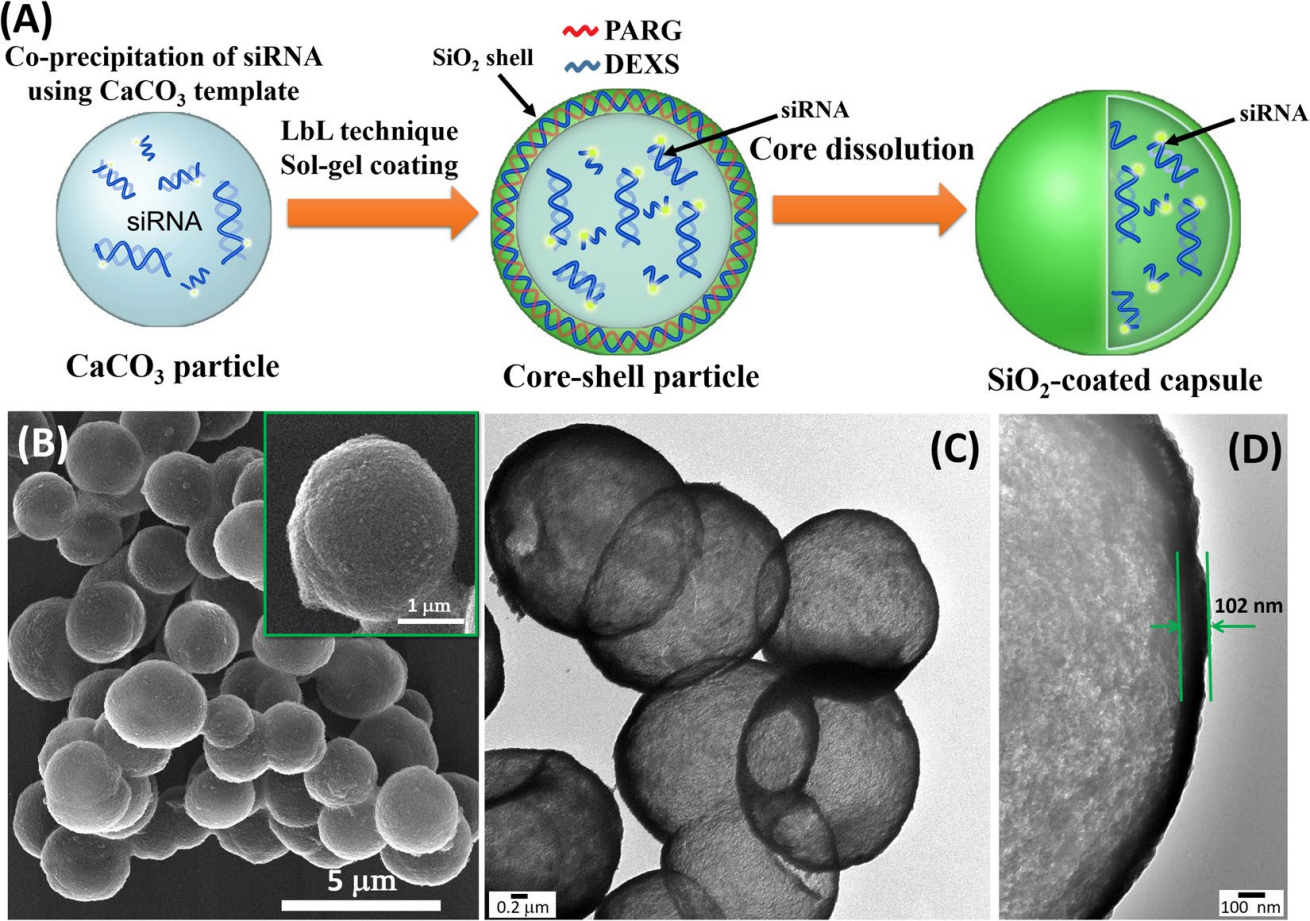
(**A**) The schematic illustration of the fabrication of SiO_2_-coated capsules and the encapsulation of antiviral siRNA. (**B**) SEM image of SiO_2_-coated capsules. (**C**,**D**) TEM images of hollow SiO_2_ -coated capsules.

Figure 1 shows the possible mechanism of antiviral siRNA delivery in the cell using hybrid microcarriers and simultaneously demonstrates the process of RNA interference for viral gene knockdown. At the first step, we have performed the modification of PE microcapsules using sol-gel chemistry (Fig. 2A). As shown in several papers^34, 40^, the integration of sol-gel method with LbL technique provides excellent opportunity to fabricate hybrid microcontainers with a drastically reduced permeability protecting bioactive molecules from premature degradation when it is internalized by the cells. To fabricate intracellular degradable capsules, we used PARG and DEXS polyelectrolytes and TEOS as sol-gel precursor to form biocompatible silica shell. After the several synthetic procedures described in the experimental section (Preparation of SiO_2_-coated capsules and encapsulation of siRNAs), hollow SiO_2_-coated capsules were obtained. Figure 2B demonstrates the morphology of SiO_2_-coated capsules after sol-gel coating. In comparison with typical PE microcapsules made of PARG and DEXS (Fig. S1), the morphology of hybrid capsules has been obviously changed: these capsules are fully covered by a dense layer of SiO_2_ nanostructure, they became robust and remained spherical shape, which is due to the enhanced mechanical properties of hybrid capsules. We further study the morphology and shell thickness of our hybrid capsules using transmission electron microscopy (TEM). As it can be clearly seen, TEM images of SiO_2_-coated capsules (Fig. 2C,D) clearly show the formation of hollow structure of composite capsules with shell thickness of ~100 nm. Importantly, the application of CaCO_3_ as template in co-precipitation process with bioactive compounds allows the *in situ* encapsulation of these substances without a loss of their bioactive properties because EDTA solution is used for dissolution of CaCO_3_^42, 43^. Whereas other templates are unable to apply because they can be removed only using calcination process, which would destroy the bioactive molecules upon high temperature. In addition, FTIR spectroscopy was used to confirm the successful SiO_2_ coating (Fig. S2). The FTIR spectrum of SiO_2_-coated capsules demonstrates the characteristic bands which are typical for SiO_2_ nanostructure: a broad band in the range of 3200–3500 cm^−1^ corresponds to the stretching vibrations of hydroxyl groups^44^. The peak at around 950 cm^−1^ can be assigned to Si–OH bending mode. The sharp peak at 1087–1095 cm^−1^ corresponds to Si–O–Si vibrations^45^. The above observation clearly shows that application of sol-gel coating does allow to fabricate well-defined spherical capsules with enhanced mechanical strength.

### Loading efficiency of siRNAs

**Figure 3:**
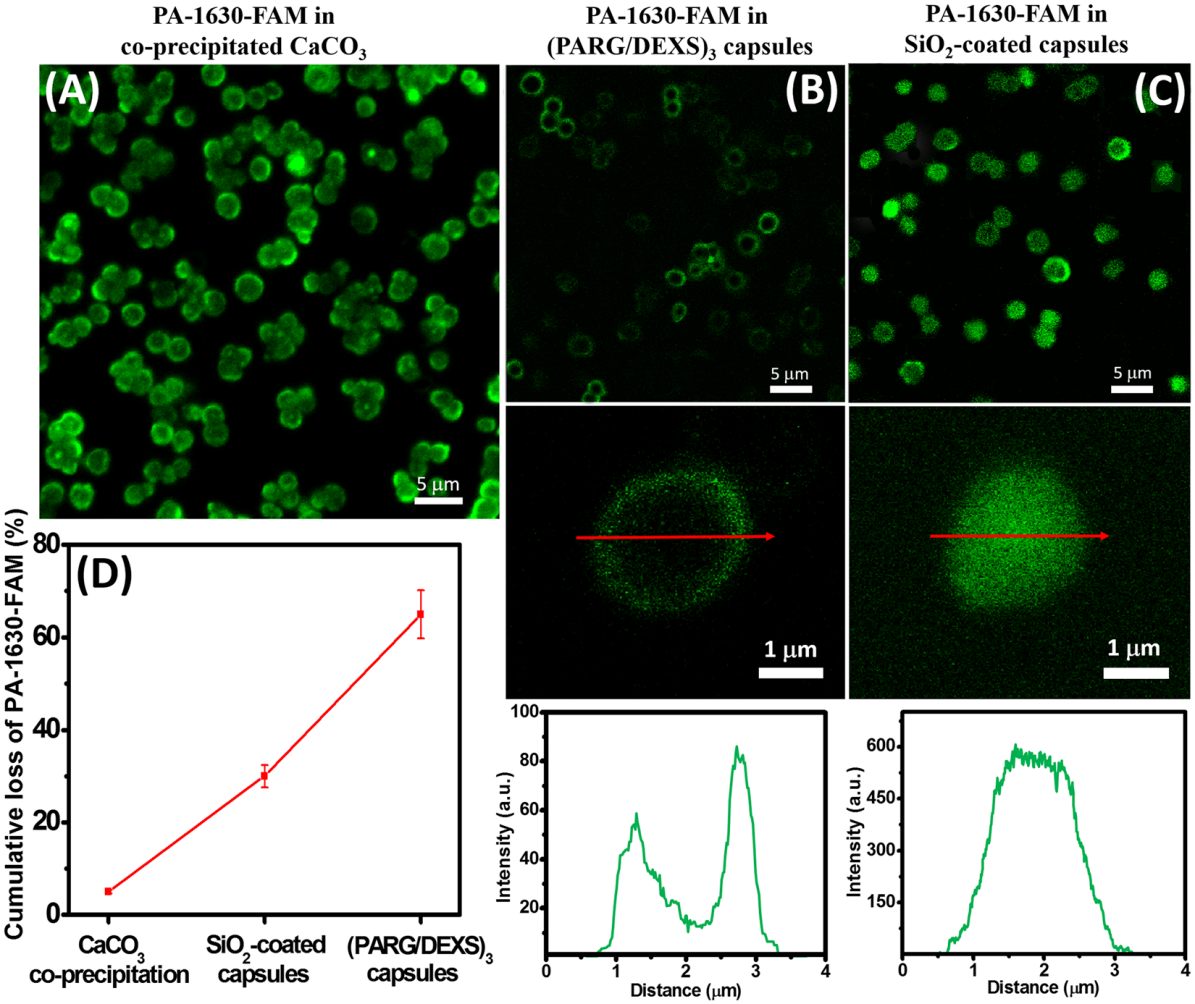
Confocal microscopy analysis of distribution of PA-1630-FAM in used carriers: (**A**) CaCO_3_ microparticles; (**B**) (PARG/DEXS)_3_ capsules; (**C**) SiO_2_-coated capsules. The plots below CLSM images are the representative fluorescence intensity profiles over the microcapsule. (**D**) Cumulative loss of PA-1630-FAM after dissolution of CaCO_3_ in SiO_2_-coated and (PARG/DEXS)_3_ capsules, respectively.

Next, we have compared the encapsulation capacity between SiO_2_-coated capsules and typical polymeric microcapsules made of PARG and DEXS. Also, we evaluate the localization of siRNA inside capsule using siRNA with fluorescent marker (PA-1630-FAM), which was monitored by confocal microscopy. Before LbL technique and sol-gel coating, we obtained the CLSM image of co-precipitated CaCO_3_ particles containing PA-1630-FAM (Fig. 3A). As it can be seen, CaCO_3_ particles containing PA-1630-FAM exhibit green fluorescence. According to the co-precipitation process, a total loading efficiency of PA-1630-FAM was ~95%. After LbL assembly of PARG and DEXS with following removal of CaCO_3_ by EDTA, we found out that (PARG/DEXS)_3_ microcapsules possess a poor green fluorescent shell (the PA-1630-FAM is localized on the polymeric shell of microcapsules) (Fig. 3B). Also the plot in panels below CLSM images of Fig. 3B represents the fluorescence intensity profile over (PARG/DEXS)_3_ microcapsule. After CaCO_3_ dissolution the major fraction of the PA-1630-FAM was released from microcapsule. The overall efficiency of encapsulated the PA-1630-FAM in (PARG/DEXS)_3_ capsules was 35% (Fig. 3D). This observation is in a good agreement with preciously reported results. M. Kakran *et al.* have also shown a low loading efficiency of RNA using polymeric capsules made of PARG and DEXS^46^. Then, we evaluated the storage capacity of hybrid capsules consisting of PARG/DEXS and SiO_2_ nanostructure. We found that SiO_2_-coated capsules can load significantly more molecules of PA-1630-FAM than (PARG/DEXS)_3_ capsules. As shown in Figs 3C and S3, PA-1630-FAM is mainly localized inside hybrid capsule. The cumulative loss of encapsulated PA-1630-FAM in SiO_2_-coated capsules was around 29%, which considerably lower than found for (PARG/DEXS)_3_ capsules (~65%). Employing sol-gel coating allows to reduce the permeability of hybrid capsules and, therefore, SiO_2_-coated capsules have ability to carry higher amount of siRNA compared to (PARG/DEXS)_3_ capsules. Introduction of silica shell reduced the pore diameter and enhanced mechanical properties of hybrid capsules, which is favorable for encapsulation of different bioactive compounds. In addition, we have confirmed the presence of encapsulated siRNA in hybrid microcapsules using acridine orange^47^. It is well known that acridine orange is a fluorescent dye that interacts with nucleic acids. In case of interaction with DNA, it exhibits an excitation maximum at 502 nm (cyan) and an emission maximum at 525 nm (green). In opposite when it binds with RNA, the excitation maximum is at 460 nm (blue) and the emission maximum is at 650 nm (red). This phenomenon is associated with electrostatic interactions occurring when the acridine molecule intercalates between the nucleic acid base pairs, and gives valuable information about the type of nucleic acid present. In our case, we observed a bright orange-to red fluorescence in the lumen of SiO_2_-coated capsules, indicating the presence of encapsulated RNA molecules inside hybrid capsules (Fig. S4). As revealed the crucial role of sol-gel coating on the increase of encapsulation efficiency of siRNAs, we then performed the encapsulation of other siRNAs (NP-1496, NP-1155 and NP-717) using LbL technique and sol-gel coating. The amount and concentration of all encapsulated siRNAs are presented in Table 1. Our data yielded that an average number of moles for all siRNA ranges from 0.41 × 10^−3^ to 0.56 × 10^−3^ pmol *per* capsule.

**Table 1:** Concentration and number of moles of siRNAs in SiO_2_-coated capsules.

Type of siRNA	Concentration of siRNA, pmol/μL	n_caps_ [pmol] (per capsule)
NP-1496 siRNA	1.80	0.41 × 10^−3^
NP-717 siRNA	2.00	0.45 × 10^−3^
NP-1155 siRNA	4.40	0.59 × 10^−3^
PA-1630-FAM	4.20	0.56 × 10^−3^

n_caps_ – number of moles of siRNA per capsule.

### Capsules-to-cell interactions and distribution of PA-1630-FAM in the cells

The levels of cytotoxicity, capsule internalization and transfection efficiency are key factors which should be considered for further *in vitro* application of our hybrid capsules for delivery of antiviral siRNAs. For this reason, fluorescent-tagged siRNA (PA-1630-FAM) was used to evaluate the capsule internalization and further cytosolic release of siRNA, as determined by CLSM. As for the study of internalization process and siRNA release, MDCK and A549 cell lines were used. MDCK and A549 cells were selected because they are two host cells easily infected by influenza A/PR/8/34.

**Figure 4:**
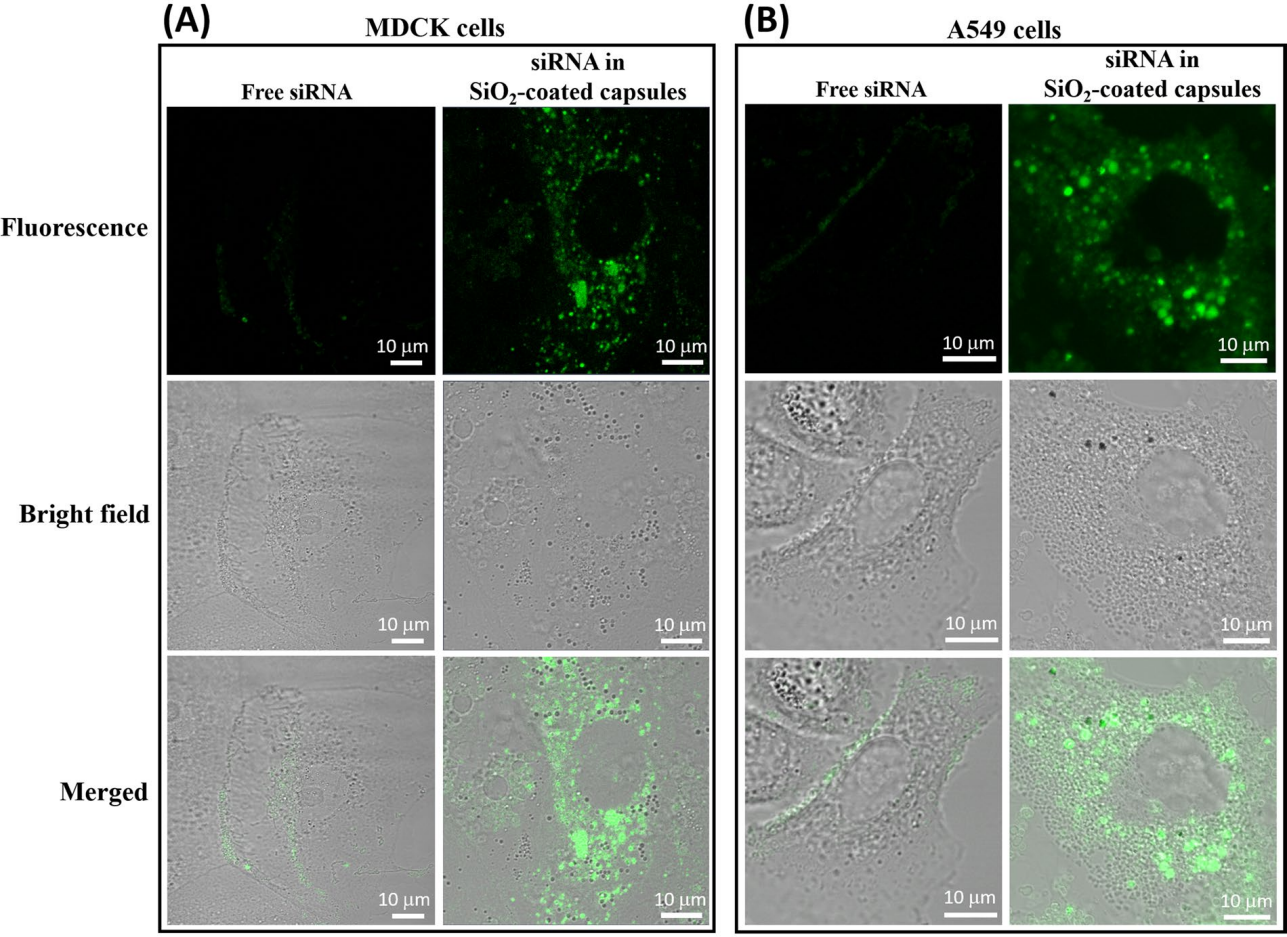
The uptake of fluorescently labeled siRNA (PA-1630-FAM) in MDCK (**A**) and A549 (**B**) cells *in vitro*. Confocal microscopy was used to compare the uptake and cellular localization of non-encapsulated and encapsulated siRNAs in SiO_2_-coated capsules after 8 h incubation with MDCK and A549 cells.

First we examined MDCK and A549 cells incubated with non-encapsulated and encapsulated siRNA (PA-1630-FAM) to confirm the enhanced cellular uptake of FAM-labeled siRNA using SiO_2_-coated capsules. Both cells showed intense cytoplasmically localized fluorescence signal after 8 h incubation with SiO_2_-coated capsules containing FAM-labeled siRNA (Fig. 4). In contrast, there was very little uptake when cells were incubated with free FAM-labeled siRNA (PA-1630-FAM) in non-encapsulated form at the same time period.

**Figure 5:**
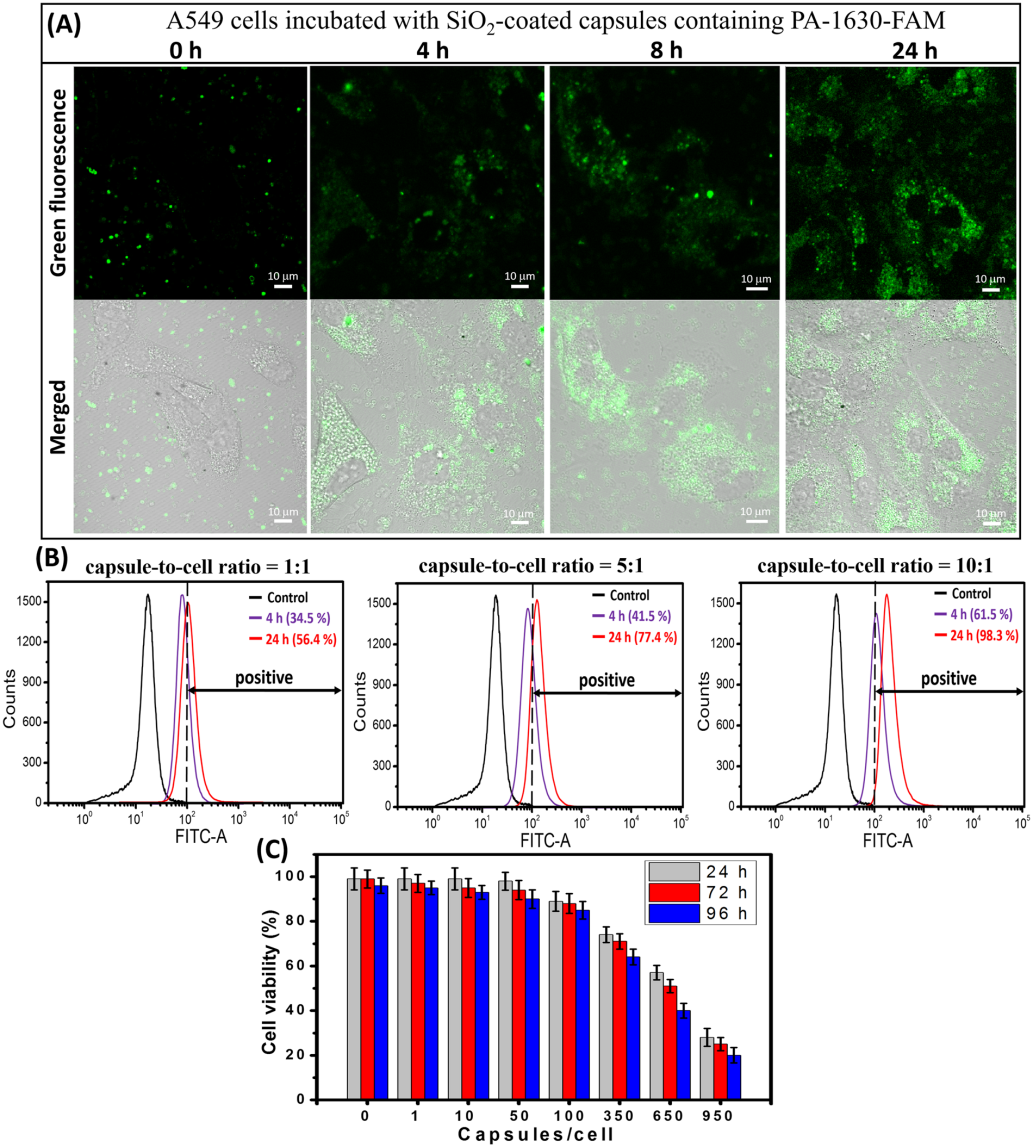
(**A**) CLSM images demonstrating the intracellular release of PA-1630-FAM from SiO_2_-coated capsules and further PA-1630-FAM distribution in A549 cells at different incubation period. (**B**) Flow cytometry analysis of SiO_2_-coated capsules uptake in A549 cells at different capsules-to-cell ratio and incubation time. (**C**) Cell viability of A549 cells incubated with SiO_2_-coated capsules at different capsules-to-cell ratios for various time intervals (24, 48, or 72 hours).

We further studied the process of capsules internalization containing fluorescently labeled siRNA (PA-1630-FAM) and siRNA release into the cellular environment. At an initial time (t = 0 h), the SiO_2_-coated capsules containing PA-1630-FAM were added to the cells at a capsules-to-cell ratio of 10:1 (Fig. 5A). It is important to mention that experiment was performed with adherent cell culture, imitating epithelial layer. At t = 0 h only green fluorescence from hybrid capsules can be observed. However, during the incubation period, the capsules mainly distributed in perinuclear compartment of the A549 cells and then began to decompose with following release of PA-1630-FAM into the cytosol of the cells. Already after 4 h incubation, the distribution of the green fluorescent spots can be observed, indicating the release of PA-1630-FAM. CLSM images recorded after 8 h clearly demonstrate the increase in homogeneous distribution of the green fluorescence of PA-1630-FAM in the cytosol. At later time point (24 h) granular intracellular distribution pattern of the green spots can be observed. At the same time, the nuclei were not stained for each time points, indicating that PA-1630-FAM cannot cross the nuclear membrane. The same tendency in capsule internalization and the cytosolic release of PA-1630-FAM were found for MDCK cells (we refer the reader to the Supporting Information, Fig. S5). Using biodegradable polymers such as polyarginine or dextran sulphate in fabrication of microcapsules would permit degradation of the microcapsule structure^46^, resulting in the formation of hollow channels and structural disorder of hybrid capsules. This process may lead to decomposition of capsule shell and further release of PA-1630-FAM. At the same time, silica shell can be degraded in the cell medium^42^. Therefore, both factors (biodegradable polymers and silica shell) leads to the *in vitro* degradation of our hybrid capsules with further release of genetic materials. We suppose that due to the ability of degradable SiO_2_-coated capsules to encapsulate active siRNA, it makes SiO_2_ shell promising for the delivery of other sensitive compounds that can be decomposed before reaching the target cells.

The cellular uptake efficiency of fluorescent SiO_2_-coated capsules was further evaluated by flow cytometry. To facilitate siRNA internalization into the cells, the experiments were performed at 37 °C for 4 and 24 h respectively. The cells (MDCK and A549) were incubated with capsules at different capsules-to-cell ratios (1:1, 5:1 and 10:1). At definite time interval, the fluorescent intensity of cells with internalized capsules was measured. As shown in Fig. 5B, at capsules-to-cell ratio of 10:1 the peak of fluorescent intensity shifted to a higher level with increasing of time incubation: the percentage of A549 cells with internalized capsules increased from 61.5% in 4 h to 98.3% in 24 h, indicating the successful internalization of SiO_2_-coated capsules. Also, we can observe that the level of capsules internalization is growing with increase of capsules-to-cell ratio: around 98.3% of cells exhibited green fluorescence at capsules-to-cell ratio of 10:1 after 24 h incubation while cellular uptake percentage was only 56.4% at capsules-to-cell ratio of 1:1 for the same incubation period. The similar trend in cellular uptake was observed in case of MDCK cells (the flow cytometry analysis for MDCK cells was presented as Supporting Information, Fig. 6S). These results clearly demonstrate that our hybrid capsules are readily internalized by both cell types. In contrast, A549 and MDCK cells treated with naked siRNA (PA-1630-FAM) show only ~1% naked siRNA uptake after 24 h incubation (Supporting Information, Fig. S7).

**Figure 6:**
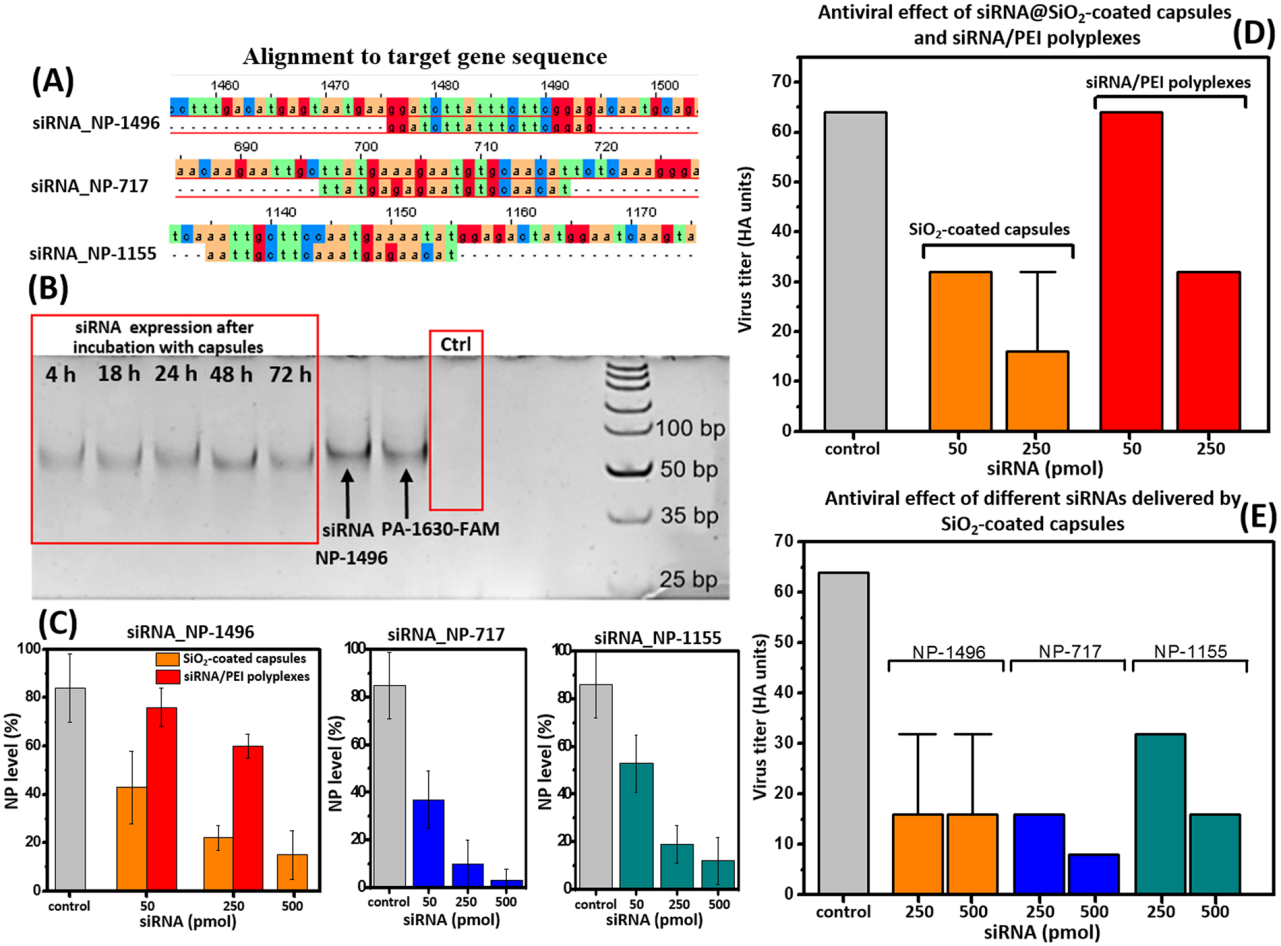
(**A**) Schematic diagrams showing the location of each siRNA in the viral genome. (**B**) Results of electrophoresis of the NP-1496 siRNA PCR products extracted from MDCK cells. (**C**) ELISA analysis showing the reduction of viral NP level in infected A549 cells treated with different anti-viral siRNAs in encapsulated form (50 pmol–1 capsule/cell; 250 pmol–5 capsules/cell; 500 pmol–10 capsules/cell). (**D**) Comparison of antiviral effect of NP-1496 siRNA delivered by siRNA/PEI polyplexes and SiO_2_-coated capsules (50 pmol–1 capsule/cell; 250 pmol–5 capsules/cell; 500 pmol–10 capsules/cell). (**E**) Inhibition of influenza virus production by different dose of siRNAs in encapsulated form. Viral titers was measured 72 h after infection in a hemagglutination assay. The data are presented as means ± standard deviation (SD) from three independent experiments. Note that some hemagglutination unit values were identical for the triplicate experiments, and so standard deviation values are 0.

The *in vitro* cytotoxicity of SiO_2_-coated capsules at different capsules-to-cell ratios was also examined for both cell types. As shown in Fig. 5C, SiO_2_-coated capsules have shown negligible effect on viability of A549 cells. Indeed, SiO_2_-coated capsules at capsules-to-cell ratios of 1:1, 1:10, 1:50 do not seriously affect the viability of A549 after 24, 72 and 96 hours incubation. At the capsules-to-cell ratio of 100:1, we observe a slight decrease in the viability of A549. The high toxic effect was only detected when the number of capsules per a cell was more than 350. The similar tendency in cell viability was observed when MDCK cells were incubated with different concentration of capsules (Fig. S8). Therefore, the previously observed high cellular uptake efficiency of SiO_2_-coated capsules can be attributed to their low cytotoxicity. Thus, we may conclude that these capsules are non-toxic and possess biodegradable properties, which is in favor to use such microcontainers for further biological implementation and delivery of different bioactive compounds, including siRNA.

### Effects of encapsulated siRNAs on the inhibition of viral nucleoprotein (NP) and influenza virus production

To determine the *in vitro* antiviral activity of three types of siRNAs (NP-1496, NP-1155 and NP-717) encapsulated inside SiO_2_-coated capsules, A549 and MDCK cells were used. The transfected cells were infected with influenza H1N1 virus. The direct ELISA analysis was used to detect viral nucleoprotein (NP) level in tested cell cultures while HA assay was employed to determine influenza virus titer in tested samples.

As we mentioned in the introduction section, the influenza A genome consists of eight RNA segments. Three of the eight RNA segments encode three components of the RNA transcriptase (PA, PB1 and PB2)^48^. The other additional RNA segments encode the nucleoprotein (NP), matrix protein, ion channel subunits, nonstructural proteins, major glycoproteins such as hemagglutinin and neuraminidase^49^. The schematic structure of influenza virus can be found in Supporting Information (Fig. S9). Recent studies have shown that NP genes play a crucial role in the virus replication, therefore, siRNAs against NP genes should inhibit influenza virus most effectively^50^. In our study, we used two new siRNAs (NP-1155 and NP-717) and one already known siRNA (NP-1496), specific for NP genes that can potentially inhibit influenza virus production. A phylogenetic alignment was used to define the conservative regions of 20–22 nucleotides that are potential targets for siRNAs in NP sequence. More than 9000 complete segment sequences of human influenza A virus NP genes were downloaded from the NCBI’s Influenza Virus Resource^51^, aligned and determined short consensus sequences with highest conservation percentage. According to Tom Tuschl’s rules we designed siRNAs (NP-1155 and NP-717) specific for viral NP genes using Dharmacon’s siRNA design algorithm. Figure 6A shows the location of each tested siRNA in the viral genome. In the blast search at National Center for Biotechnology Information (http://blast.ncbi.nlm.nih.gov/Blast.cgi), two siRNAs (NP-1155 and NP-717) are novel and none of them were found sequence homology with human genome or any other host genome. Only NP-1496 siRNA has been already tested for influenza A virus inhibition and was shown as good silencer of NP gene and virus inhibitor^48^.

Employing direct ELISA analysis, three types of siRNAs encapsulated inside SiO_2_-coated capsules (the concentration of each encapsulated siRNA in capsules is shown in Table 1) were examined if they can reduce viral NP level in infected cells. For this experiment, SiO_2_-coated capsules containing antiviral siRNA were introduced into the suspension of cells (~1 × 10^5^) to generate the systems with 50, 250 and 500 pmol of encapsulated siRNA respectively. After 24 h incubation the cells were washed with PBS to remove non-uptake capsules and then were infected with H1N1 A/PR/8/34 virus at an infectious doze (moi) of 0.01. At 72 h post-infection, culture supernatants were harvested to determine the virus NP level. As for negative control, the cells without incubation with capsules were used. The presence of encapsulated siRNA transfected into the cells during the incubation period (from 4 to 72 h) was confirmed by gel-phoresis (Fig. 6B). For all time points siRNA is stable and not degraded during the incubation process. The results of ELISA analysis showed that the amount of viral NP production in cells incubated with SiO_2_-capsules containing antiviral siRNAs is remarkably lower than for the control cells (Fig. 6C). The viral NP production decreased at different dose of siRNAs. It indicates that siRNAs delivered via SiO_2_-coated capsules affect virus protein synthesis. For comparison, ELISA experiment was conducted with siRNA/PEI polyplexes containing the same amount of NP-1496 siRNA (Fig. 6C). The resulting viral NP level was higher in comparison with SiO_2_-coated capsules at an equivalent concentration of NP-1496 siRNA, confirming the vital role of SiO_2_-coated capsules in delivery of antiviral siRNA affecting on virus protein synthesis.

The next experiment was conducted using HA assay to demonstrate that the siRNA delivered by SiO_2_-coated capsules was superior to siRNA/PEI polyplexes. The NP-1496 siRNA was chosen for this experiment because of a high potency of NP-1496 siRNA in inhibiting influenza virus production as it was previously demonstrated^48^. In this experiment, NP-1496 siRNA (50–250 pmol) delivered by SiO_2_-coated capsules was compared with siRNA/PEI polyplexes in terms of their ability to inhibit H1N1 replication in A549 cells (1 × 10^5^). The siRNA delivery was achieved before the virus infection. At 72 h after virus infection, the cell pellets was used to determine the virus titer. The A549 cells with H1N1 influenza virus (cells + virus) was used as a negative control. As it can be clearly seen in Fig. 6D, the NP-1496 siRNA delivered by SiO_2_-coated capsules reduced the viral titer more effectively compared to the NP-1496 siRNA/PEI polyplexes delivery method. These findings result in an enhanced antiviral effect of NP-1496 siRNA in inhibiting H1N1 influenza virus titer in A549 cells. Also, the therapeutic potency of antiviral siRNA delivery by SiO_2_-coated capsules has been shown on the example of MDCK cells. (Supporting Information, Fig. S10 and Table S1).

Then, we compared the antiviral activity of three siRNAs (NP-1155, NP-717 and NP-1496) delivered by SiO_2_-coated capsules (Fig. 6E). According to the previous observation, the optimum amount of siRNA was 250–500 pmol for 1 × 10^5^ cells^48^. In case of NP-1496 siRNA-SiO_2_-coated capsules, we can observe 2-fold decrease in virus titer compared with control (cells + virus). The NP-1155 siRNA demonstrates 4-fold decrease in virus titer. The transfection with NP-717 siRNA-SiO_2_-coated capsules was the most effective, resulting in 8-fold decrease of virus titer. Among tested siRNAs, two novel siRNAs (NP-1155 and NP-717) demonstrated the inhibition of virus production in dose-dependent manner.

The siRNAs delivery using hybrid microcarriers has the considerable therapeutic potential for treating and prevention of influenza virus infection. We suppose that application of hybrid microcarriers for *in vitro* delivery of anti-viral siRNAs can be considered as more promising approach than well-known physical methods of RNA delivery such as electroporation. Although electroporation has been used for *in vitro* delivery of anti-viral siRNA into the cells, leading to inhibition of viral replication^48^, but this method often leads to cell death since the electrical fields cause permanent permeable state of the membrane and, hence, the consequent loss of cell homeostasis^52^. As shown, our capsules possess very low toxicity and, therefore, can be considered as more promising delivery vehicles in comparison with PEI/siRNA polyplexes is significantly more toxic than our capsules. Although, PEI/siRNA polyplexes can also deliver siRNA resulting in the reduction of virus production. But, the further increase of concentration of PEI/siRNA polyplexes in order to generate high amount of siRNA leads to significant reduction of the cell viability: it requires more than 2 ng/μL of PEI for loading 500 pmol of siRNA which is highly toxic (Supporting Information, Fig. S11). Therefore, it limits the application of PEI/siRNA polyplexes for clinical implementation.

## Conclusions

In this study, we demonstrated that hybrid microcarriers made of SiO_2_ nanostructures and polypeptides/polysaccharides provides a significant increase in encapsulation efficiency of siRNA. The Advantage in combination of the unique structural properties of SiO_2_ with LbL technique paves an efficient strategy platform for the fabrication of stable and safe microcontainers with a low permeability and, protecting from premature decomposition of siRNA before reaching the target cells. Due to the biodegradable ability of obtained hybrid capsules, the siRNA can release into the cytosol of the cells after capsules internalization. *In vitro* experiments have also confirmed that these capsules are not toxic, producing the high local concentration of siRNA in the cells. This siRNA delivery system was proved to work for both cell lines (MDCK and A549). Anti-viral activity of two novel siRNAs (NP-1155 and NP-717) and NP-1496 siRNA using SiO_2_-coated capsules was tested to successfully serve as viral carriers for the *in vitro* delivery. We found that NP-717 effectively reduced the NP as well as virus production. Other siRNAs (NP-1155 and NP-1496) also show some inhibitory effect after virus infection. These findings have significant implications for the use of microencapsulation technology for effective delivery of siRNAs in therapy of influenza virus infection. The new RNAi delivery system will allow us to follow up further *in vivo* investigations.

## Methods

### Materials

Poly-L-arginine hydrochloride (PARG, MW > 70 000), Dextran sulfate (DEXS, MW > 500 000), Tetraethyl orthosilicate (TEOS, MW = 208.33, 99.9%), Polyethylenimine (PEI, MW ~25 000), calcium chloride dehydrate, anhydrous sodium carbonate, ethylenediaminetetraacetic acid trisodium salt (EDTA) were obtained from Sigma-Aldrich and used without further purification. Absolute ethanol (C_2_H_5_OH, 98%) was used in sol-gel synthesis. Deionized (DI) water with specific resistivity higher than 18.2 MΩcm from a three-stage Milli-Q Plus 185 purification system was used.

### siRNAs

All RNA oligonucleotides were synthesized by “DNA-synthesis” (Moscow, Russian Federation). The sequences of all tested siRNAs were the following: NP-1496 siRNA (sense 5′-ggAUCUUAUUUCUUCggAgdTdT-3′; antisense 5′-CUCCgAAgAAAUAAgAUdTdT-3′); NP-717 siRNA (sense 5′-UUAUgAgAgAAUgUgCAACAU-3′; antisense 5′-gUUgCACAUUCUCUCAUAAgC-3′); NP-1155 siRNA (sense 5′-AAUUgCUUCAAAUgAgAACAU-3′; antisense 5′-gUUCUCAUUUgAAgCAAUUUg-3′). In addition, the modified RNA oligonucleotides NP-1496-1/2, in which the 2_-hydroxyl group was replaced into a 2_-O-methyl group in every nucleotide residue, and single strand PA-1630-FAM, containing carboxyfluorescein (FAM) on its 3′-end, were also synthesized by “DNA-synthesis”. The sequence of PA-1630-FAM with fluorescence marker was the following: sense 5′-UgAgCCACACAAAUgggAA-3′; antisense 5′-UCCCAUUUgUgUggCUCUU-*FAM-3′. The oligonucleotides were dissolved in depcH2O (100 uM), aliquoted and stored at −20 °C. For experimental exposure required amount of complementary single strand oligonucleotides were mixed and annealed by heating first to 95 °C for 5 min, then hybridized in the temperature mode: slowly reducing the temperature by 1 °C every 30 sec until 35 °C, then by 1 °C every 1 min until 5 °C. The resulting siRNA duplexes were analyzed by 15% PAAG electrophoresis.

### Preparation of SiO_2_-coated capsules and encapsulation of siRNAs

The CaCO_3_ microparticles were used as cores and prepared according to the previously reported method^53^. Then, CaCO_3_ particles were suspended in an aqueous solution of PARG (1 mg/mL) containing 0.15 M NaCl. The PARG was allowed to adsorb onto CaCO_3_ particles for 10 min. Next, the dispersion was centrifuged and washed three times with deionized water to remove non-adsorbed polyelectrolyte. After that, the deposition of DEXS was carried out on the PARG-coated CaCO_3_ particles using the same manipulation. Such procedure of PARG/DEXS assembly was repeated several times in order to form core-shell structure with following shell architecture: (PARG/DEXS)_2_ + PARG^54^. Termination of capsule preparation by PARG is very important due to further SiO_2_ coating. Afterwards, core-shell particles were centrifuged and washed three times in deionized water. In the second step, sol-gel approach was applied for SiO_2_ coating. Before sol-gel synthesis, core-shell particles were dispersed in 4 mL of ethanol and ultrasonically treated for 3 min in ultrasonic bath. Then, this suspension was mixed with 17.5 mL of water-ethanol solution (77%) and 100 μL of TEOS was added. The obtained mixture was stirred for 20 min with following addition of 200 μL of ammonia solution (25%). The final suspension was stirred for 3 hours at room temperature. Finally, the sample was centrifuged and washed three times with deionized water. The dissolution of CaCO_3_ template was accomplished by exposure of the sample to 0.2 M EDTA aqueous solution. Finally, hollow SiO_2_-coated capsules were fabricated. Typically obtained amounts of SiO_2_-coated capsules from one synthesis were 4.4 ÷ 7.4 × 10^6^ capsules.

To perform *in situ* encapsulation procedure of siRNAs, 120 μL of used siRNA (PA-1630-FAM, NP-1496, NP-717 and NP-1155) with concentration of 100 pmol/μL has been co-precipitated with CaCO_3_ particles following the multilayer assembly of PARG/DEXS. This procedure is similar to already reported on nucleic acid encapsulation via CaCO_3_ templating^55^. In our work, the obtained core-shell particles containing siRNA molecules were used for sol-gel coating as described above. The encapsulation efficiency of siRNAs was measured by absorbance at 260 and 280 nm using the Nanodrop 2000 Spectrophotometer. The final amount of each encapsulated siRNA are enlisted in Table 1. The cumulative loss of PA-1630-FAM was calculated as the difference between the initial amount of siRNA and the amount of siRNA in the supernatant after core dissolution.

### Characterization of SiO_2_-coated capsules

The morphology of SiO_2_-coated capsules was observed on SUPRA 55VP Scanning Electron Microscope (SEM ZEISS, Germany) at an accelerating voltage of 15 kV. Before SEM analysis, the diluted ethanol suspension of SiO_2_-coated capsules was dropped on a glass slide, air dried, and coated with gold (Au) before observation. Further, the morphology and shell thickness were studied using a JEOL JEM-2100F transmission electron microscope (TEM). The SiO_2_-coated capsules were also observed using LSM 880 Confocal Laser Scanning Microscope, Carl Zeiss, Germany.

### Viruses and titer assays

Influenza virus subtype H1N1 A/PR/8/34 (PR8) from Influenza Research Institute collection (St-Petersburg, Russia) were grown in the allantoic cavity of 10-day-old embryonated chicken at 37 °C. Allantoic fluid was harvested 48 h after virus inoculation and stored at −80 °C. Virus titer was measured by hemagglutination assay, using 50% tissue culture infective dose (TCID50) assay. Serial 10-fold dilutions of allantoic fluid were added onto a monolayer of MDCK and A549 cells in 96-well culture plates and incubated for three days. The viral titer for each sample was calculated by the Reed–Muench method^56^.

The hemagglutination assay (HA) was carried out in U-bottom 96-well plates in order to evaluate the virus titer. Serial 2-fold dilutions of supernatants were mixed with an equal volume of a 0.5% suspension (vol_vol) of chicken erythrocytes and incubated on ice for 30 min. Wells containing an adherent, homogeneous layer of erythrocytes were scored as positive.

### Cell Culture and incubation of the cells with SiO_2_ -coated capsules

Madine-Darby canine kidney (MDCK) and human lung epithelial (A549) cells were kindly provided by the colleagues from Influenza Research Institute collection (St-Petersburg, Russian Federation). Cells were cultivated in cultural flasks using Dulbecco’s Modified Eagle Medium (DMEM) (Lonza, Switzerland); supplemented with 100 IU/ml penicillin, 0.1 mg/ml streptomycin (Biolot, Russia), and 10% Fetal Bovine Serum (FBS) (Hyclone, USA). After achievement of confluence (>80%), cells were detached with trypsin/EDTA solution (Invitrogen, USA) for 10 min and were seeded on cultural flasks at a density of 5.0 × 10^3^ cells/cm^2^.

We encapsulated PA-1630-FAM in order to monitor the release behavior of bioactive molecules from SiO_2_-coated capsules in the cells using CLSM. The capsules with PA-1630-FAM were added to suspensions of A549 and MDCK cells at a capsule-to-cell ratio of 10:1 and seeded in culturing flasks at a density of 5,0 × 10^3^ cells/cm^2^ at 37 °C for 4, 8 and 24 h. At definite time points, the cells were viewed under CLSM (Carl Zeiss, Germany) with 40x and 63x high numerical-aperture oil immersion objectives. The uptake behavior of SiO_2_-coated capsules containing PA-1630-FAM by MDCK and A549 cells was evaluated using flow cytometry (FACS Aria, BD, USA). The hybrid capsules were incubated with the cells for 24 at different capsules-to-cell ratios (1:1, 5:1 and 10:1).

### Virus Infection

For siRNAs introduction, the SiO_2_-coated capsules containing anti-viral siRNA (NP-1496, NP-1155 or NP-717) were added to cells in the monolayer in the 24x well plate (10^5^ cells per well). After 24 h incubation, the cells were washed with PBS solution to remove non-uptake capsules. Then, 0.01 moi of PR8 virus in infection medium αMEM + L-glutamine, consisting trypsin TPCK 0.1 ug/ml and 100 ug/ml streptomycin was added to each well. After 1 h incubation at 8 °C, the infectious medium was washed and the same medium without virus was added. The cells were cultured at 37 °C under 5% CO_2_. At definite time period after infection (72 hours), the supernatants were harvested from infected cultures and the virus titer was determined.

### Cell viability assay

The SiO_2_ -coated capsules were added and incubated with MDCK and A549 cells for 24 h. The cell viability was defined by differential counting in hemocytometer using vital staining with trypan blue. Briefly, after 24 hours incubation, culture medium that contained detached cells were harvested, adherent fraction of cells were detached by trypsin/EDTA solution and mixed with previously described portion of culture medium, so the all fractions of cells were represented in further analysis. For analysis, 20 μL of cell suspension were mixed with same amount of 0, 4% trypan blue (Vecton, Russia) solution. Stained cells were analyzed *ex temporo,* cells free of dye were counted as viable.

### Direct enzyme-linked immunosorbents assays (ELISA)

After 72 hours post infection, the supernatants were removed from the cells. The viral nucleoprotein (NP) level was determined using direct ELISA analysis with streptavidin-HRP-conjugated mouse antibody targeted viral NP (prepared by Influenza Research Institute). The cells were fixed with 80% cold acetone for 1 h and washed three times with phosphate buffered saline with 0.05% Tween (PBST), then incubated with 5% milk dissolved in PBST, washed three times. A streptavidin-HRP-conjugated mouse anti-NP antibody was added to each well at 1:5000 concentration and incubated at 37 °C for 1 h. Immunoreactivity was analyzed by adding to the samples TMB Peroxidase EIA Substrate Kit (Bio-Rad) according to manufactures protocols. Absorbance correlated with amount of target nucleoprotein in cells was measured on multifunctional reader CLARIOstar®(BMG LABTECH, Germany).

### RNA isolation, reverse-transcription, and quantitative real-time-polymerase chain reaction (qRT-PCR)

To confirm the presence of siRNA in the cells, we used qRT-PCR. The MDCK cells after incubation with SiO_2_-coated capsules containing siRNA in 6x well plate were harvested with trypsin-EDTA solution and miRNA fraction was extracted using the mirVana™ miRNA Isolation Kit (Invitrogen, USA) according to the manufacturer’s instructions. Then extracted RNA was treated with mirVana™ qRT-PCR miRNA Detection Kit (Invitrogen, USA) according to the manufacturer’s instructions.

## Electronic supplementary material

Supporting info
