# Supplementary material for: Hybrid inorganic-organic capsules for efficient intracellular delivery of novel siRNAs against influenza A (H1N1) virus infection
Source: Sci Rep. 2017 Mar 7;7:102. doi: 10.1038/s41598-017-00200-0 (PMC5427965; doi:10.1038/s41598-017-00200-0)
Supplement: Supplementary file 1 — Supporting info [file 41598_2017_200_MOESM1_ESM.pdf]

Supporting Information

Hybrid inorganic-organic capsules for efficient intracellular delivery of novel siRNAs against influenza A (H1N1) virus infection

Alexander S. Timin[1*] , Albert R. Muslimov[2,5], Aleksandra V. Petrova[3], Kirill V. Lepik[2], Maria V. Okilova[2], Andrey V. Vasin[3,4], Boris V. Afanasyev[2], Gleb B. Sukhorukov[1,5,6*]

[1]RASA center in Tomsk, Tomsk Polytechnic University, Lenin Avenue, 30, 634050, Tomsk, Russian Federation.

[2]First I. P. Pavlov State Medical University of St. Petersburg, Lev Tolstoy str., 6/8, 197022, Saint-Petersburg, Russian Federation.

[3]Research Institute of Influenza, Popova str., 15/17, 197376, Saint-Petersburg, Russian Federation.

[4]Department of Molecular Biology, Peter The Great St. Petersburg Polytechnic University, Polytechnicheskaya, 29, 195251, St. Petersburg, Russian Federation.

[5]RASA center in St. Petersburg, Peter The Great St. Petersburg Polytechnic University, Polytechnicheskaya, 29, 195251, St. Petersburg, Russian Federation.

[6]School of Engineering and Materials Science, Queen Mary University of London, Mile End Road, London E1 4NS, United Kingdom. Correspondence and requests for materials should be addressed to A. S. Timin (e-mail: a_timin@mail.ru) and G. B. Sukhorukov (e-mail: g.sukhorukov@qmul.ac.uk)

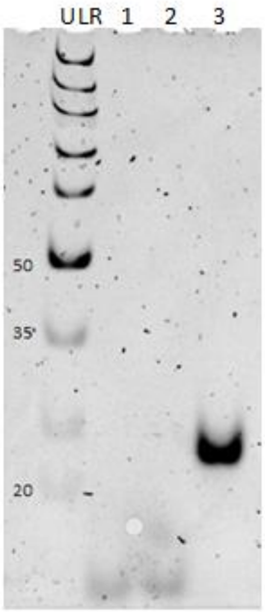

15% PAAG electrophoresis: ULR-ultra low range DNA ladder (Fermentas);
1-NP_717-1 (5’ single strand RNA UUAUgAgAgAAUgUgCAACAU);
2-siRNA_NP717-2 (3’ single strand RNA gUUgCACAUUCUCUCAUAAgC);
3-dubblestrand siRNA_NP-717 after hybridization.

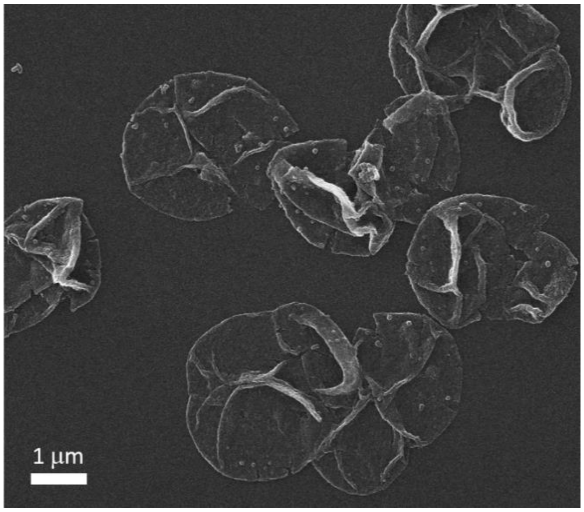

Figure S1. SEM image of PARG/DEXS microcapsules.

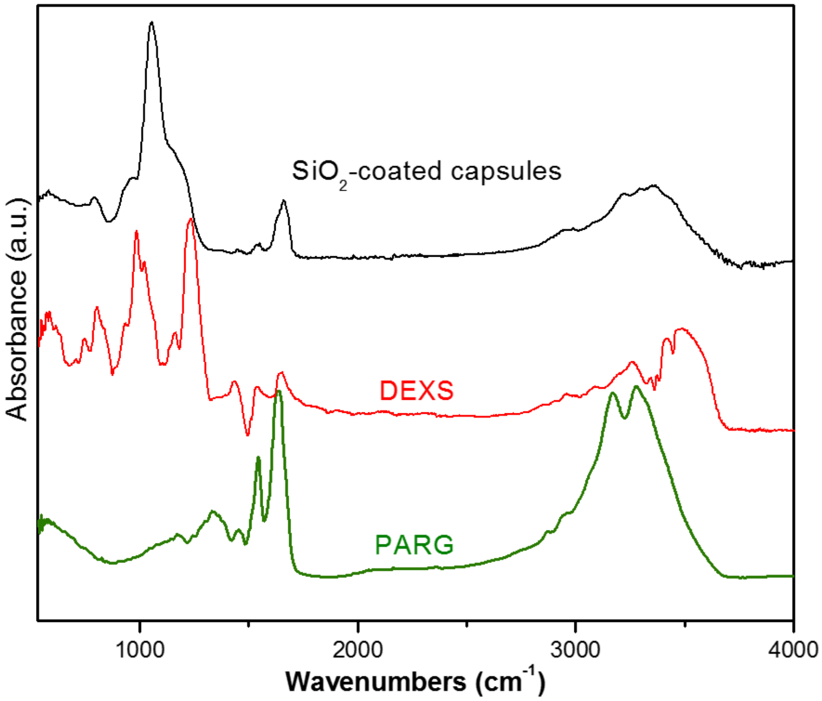

Figure S2. FTIR spectra of SiO_2 -coated capsules, DEXS and PARG.

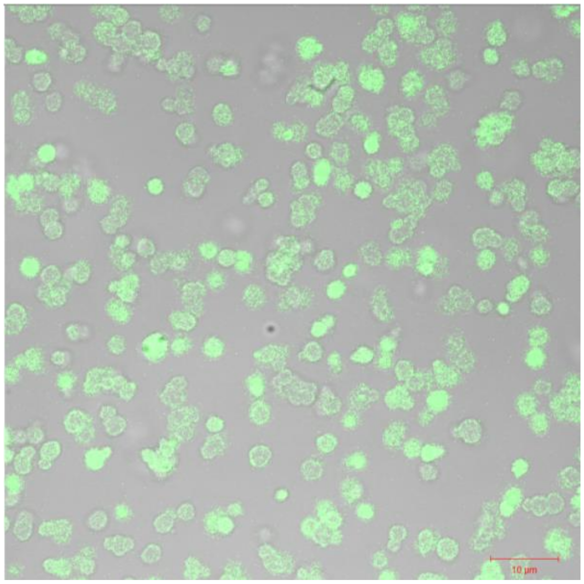

Figure S3. CLSM image of SiO_2-coated capsules with PA-1630-FAM after dissolution of $CaCO_3$

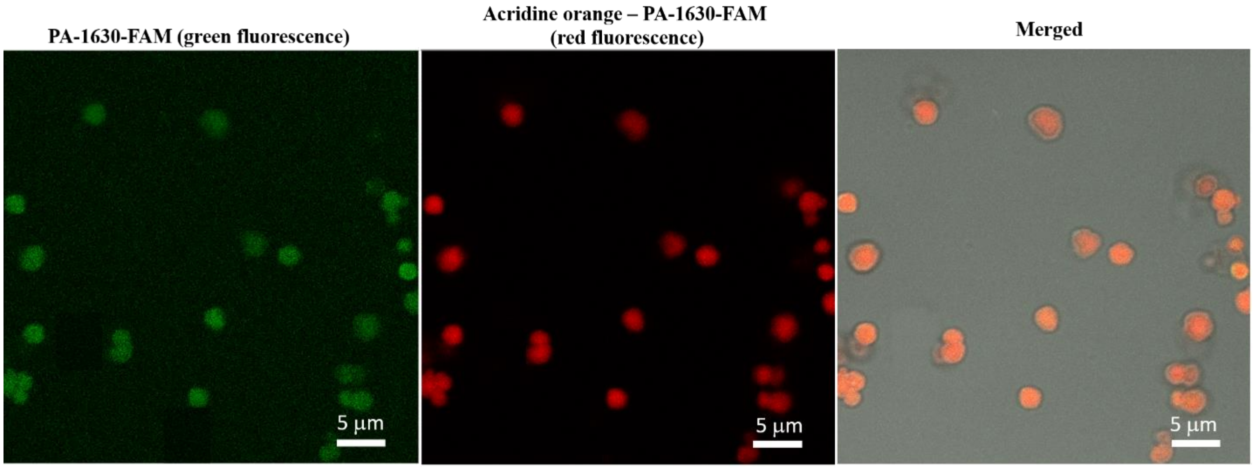

Figure S4. CLSM images of SiO_2-coated capsules with PA-1630-FAM, interacting with acridine orange.

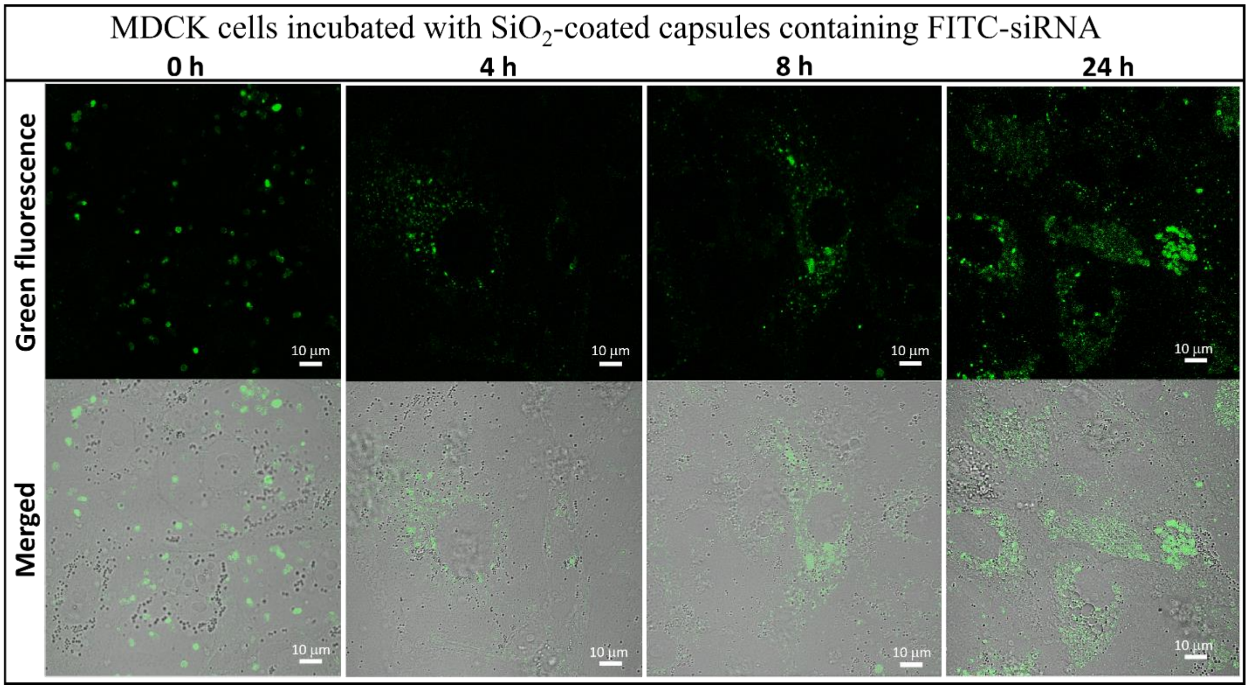

Figure S5. CLSM images demonstrating the intracellular release of PA-1630-FAM from SiO_2-coated capsules and further PA-1630-FAM distribution in MDCK cells at different incubation period.

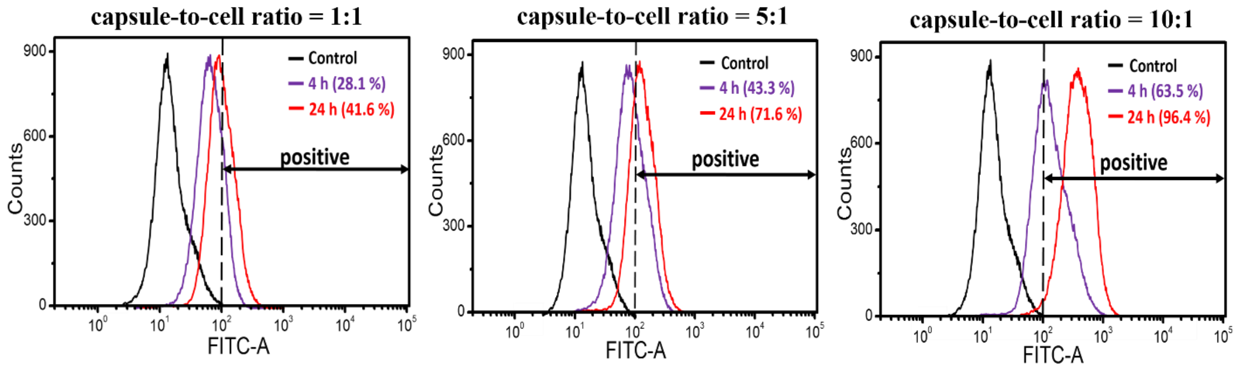

Figure S6. Flow cytometry analysis of SiO_2-coated capsules uptake in MDCK cells at different capsules-to-cell ratio and incubation time.

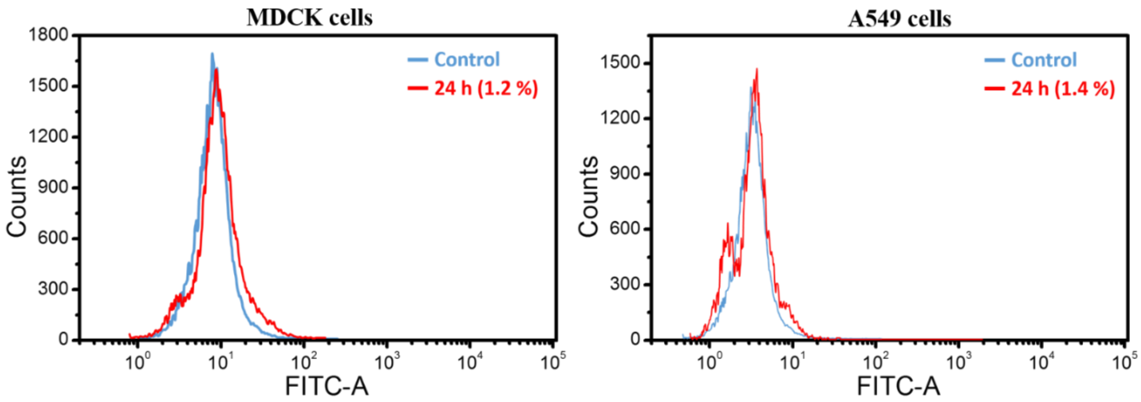

Figure S7. Flow cytometry analysis of free siRNA uptake in MDCK and A549 cells.

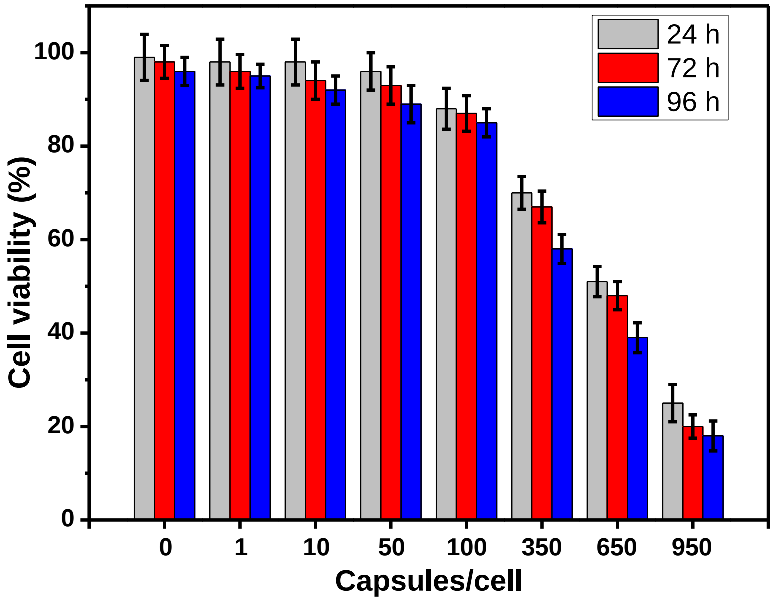

Figure S8. Cell viability of MDCK cells incubated with SiO_2-coated capsules at different capsules-to-cell ratios for various time intervals (24, 48, or 72 hours).

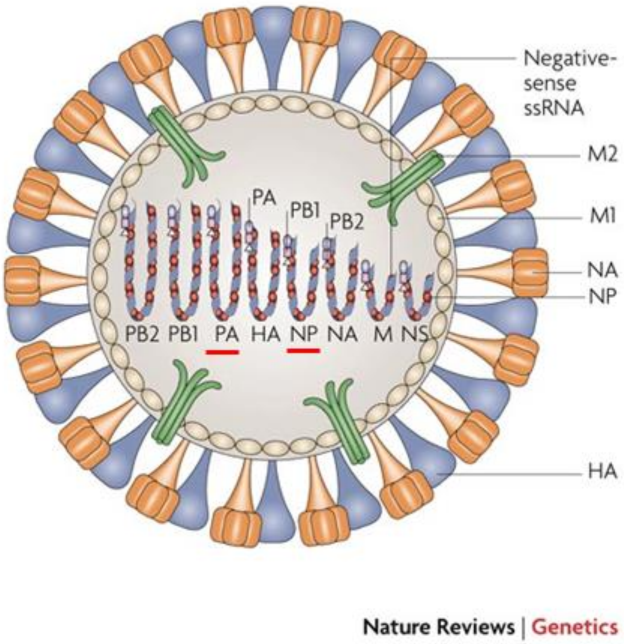

Figure S9. Simplified schematic structure of influenza virus target genes are red underlined. Nature Reviews Immunology 7, 267-278 (April 2007)

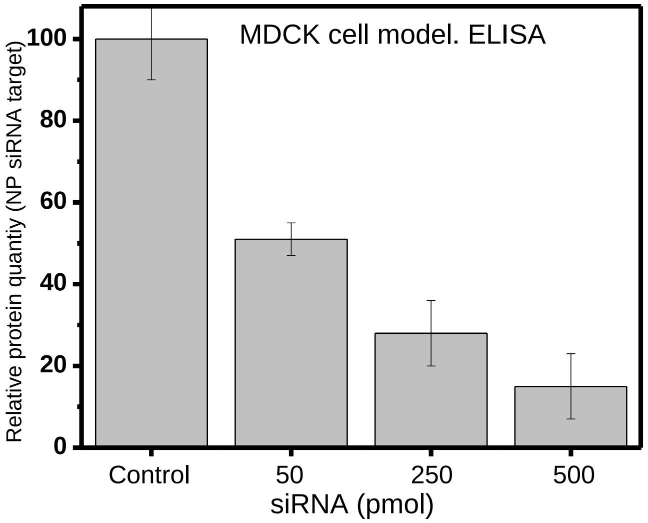

Figure S10. ELISA analysis showing the reduction of viral NP level in infected MDCK cells treated with different anti-viral NP-1496 siRNA in encapsulated form.

Table 1. Effects of siRNAs in encapsulated form on influenza virus production in MDCK and A549 cells

Virus production (titer in HA units)			
**Exp.**	**siRNA in encapsulated form**	**A549 cells**	**MDCK cells**
**1**	Virus control	64	256
	NP-1496, 250 pmol	32	32
	NP-1496, 500 pmol	32	32
**2**	Virus control	64	-
	NP-717, 250 pmol	16	-
	NP-717, 500 pmol	8	-
**3**	Virus control	64	-
	NP-1155, 250 pmol	32	-
	NP-1155, 500 pmol	16	-
	NP-1496, PEI	32	64

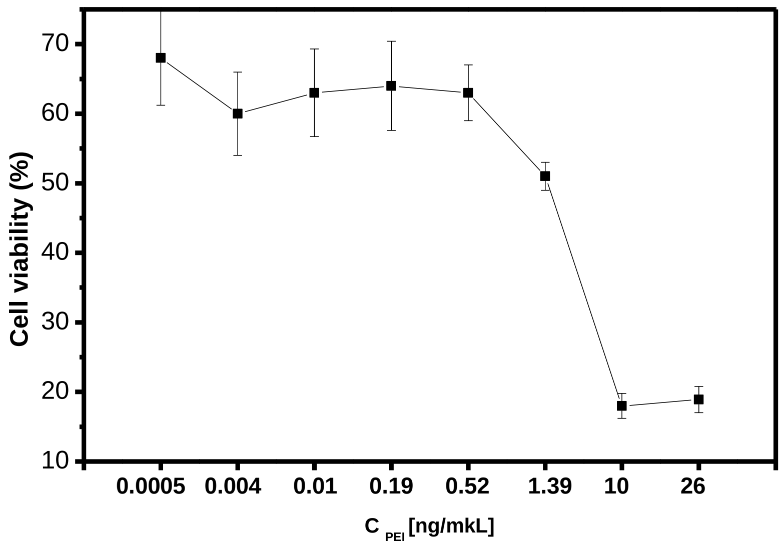

Figure S11. Reduction in the viability of MDCK cells caused by using PEI.
